# Metagenomic Characterization of the *Maerua crassifolia* Soil Rhizosphere: Uncovering Microbial Networks for Nutrient Acquisition and Plant Resilience in Arid Ecosystems

**DOI:** 10.3390/genes16030285

**Published:** 2025-02-26

**Authors:** Sumayah M. Alharbi, Nadiah Al-Sulami, Hadba Al-Amrah, Yasir Anwar, Ola A. Gadah, Lina Ahmed Bahamdain, Mohammed Al-Matary, Amnah M. Alamri, Ahmed Bahieldin

**Affiliations:** Department of Biological Science, Faculty of Science, King Abdulaziz University, Jeddah 21589, Saudi Arabiahggaber@kau.edu.sa (H.A.-A.); yanwarulhaq@kau.edu.sa (Y.A.); malmatry0002@stu.kau.edu.sa (M.A.-M.); aalomri@kau.edu.sa (A.M.A.);

**Keywords:** drought, metagenomic, plant growth-promoting, relative abundance, rhizobiome

## Abstract

**Background/Objectives:***Maerua crassifolia*, a threatened medicinal species endemic to drylands, exhibits a pronounced drought sensitivity. Despite the critical role of microorganisms, particularly bacteria and fungi, the microbial consortia in *M. crassifolia’s* rhizosphere remain underexplored. **Methods:** Metagenomic whole genome shotgun sequencing (WGS) was employed to elucidate the taxonomic composition of bacterial and fungal communities inhabiting the soil rhizosphere of *M. crassifolia*. **Results:** The data revealed a marked predominance of bacterial genomes relative to fungal communities, as evidenced by non-redundant gene analysis. Notably, arbuscular mycorrhizal fungi (AMF), specifically *Rhizophagus clarus*, *Rhizophagus irregularis* and *Funneliformis geosporum*, are key rhizosphere colonizers. This study confirmed the presence of phosphate-solubilizing bacteria (PSB), such as *Sphingomonas* spp., Cyanobacteria and Pseudomonadota, underscoring the critical role of these microorganisms in the phosphorus cycle. Additionally, the study uncovered the presence of previously uncharacterized species within the phylum Actinobacteria, as well as unidentified taxa from the Betaproteobacteria, Gemmatimonadota and Chloroflexota phyla, which may represent novel microbial taxa with potential plant growth-promoting properties. **Conclusions:** Findings suggest a complex, symbiotic network where AMF facilitate phosphorus uptake through plant–root interactions. In a tripartite symbiosis, PSB enhance inorganic phosphorus solubilization, increasing bioavailability, which AMF assimilate and deliver to plant roots, optimizing nutrition. This bacterial–fungal interplay is essential for plant resilience in arid environments. Future investigations should prioritize the isolation and characterization of underexplored microbial taxa residing in the rhizosphere of *M. crassifolia*, with particular emphasis on members of the Actinobacteria, Betaproteobacteria, Gemmatimonadota and Chloroflexota phyla to uncover their roles in nutrient acquisition and sustainability.

## 1. Introduction

*Maerua crassifolia*, commonly referred to as Sarh, is a perennial, drought-resistant plant belonging to the Capparaceae family [1,2]. Its leaves are rich in minerals such as calcium and iron, and amino acids such as phenylalanine, tyrosine, linoleic and alpha-linolenic acids, which serve as a valuable food source in low-income areas [3,4]. Traditionally, this plant has been used to treat various ailments due to its medicinal properties [5,6,7,8,9,10,11]. *M. crassifolia* is now considered endangered, prompting conservation efforts. A detailed micropropagation protocol has been developed for the in vitro culture of the species [12], but further strategies are still needed to support sustainable agricultural practices within traditional communities.

Drought, one of the most important environmental challenges, significantly limits plant growth and poses a major obstacle to achieving sustainability [13]. Plant resistance to drought and growth can be enhanced by the rhizobiome [14]. Among these rhizosphere-dwelling microorganisms are plant growth-promoting microorganisms (PGPMs), which have been demonstrated to improve plant stress tolerance and facilitate growth [15]. The rhizosphere serves as a notable interface facilitating the exchange of resources between plants and microorganisms [16,17,18]. This interaction is primarily initiated by root exudation, the main communication pathway between plants and soil microorganisms. After this initial chemical signaling, a complex sequence of exchanges involving metabolites, molecular signals and nutrients takes place. The symbiotic associations observed in the rhizosphere stem from this intricate network of below-ground interactions [19,20].

Using bacterial and fungal inocula in combination with organic amendments is a viable way to incorporate nutrient management methods into soils that have deteriorated [21,22]. These inoculums may improve soil fertility by moving, relocating, mineralizing and using important nutrients including phosphorus (P), potassium (K) and iron (Fe). They also help with the buildup of organic matter and the fixation of nitrogen (N) from the atmosphere [23,24,25]. According to previous research, arbuscular mycorrhizal fungi (AMF) and nitrogen-fixing bacteria are responsible for 5–20% of the total annual nitrogen requirement in grasslands and savannahs. In temperate and boreal forests, AMF give up to 80% of the needed N, whereas bacteria and fungi combined assist 75% of total P uptake by plants [26]. The key methods by which bacteria and fungi promote nutrient bioavailability are N fixation and the mobilization of P, K and Fe via the formation of organic acids and siderophores. In addition, these microorganisms release organo-polysaccharides and proteins, including glomalin, mucilages and hydrophobins. These substances are important for enhancing the stability of soil aggregates [23,24,27].

The metagenome, the genetic material within an environment [28], is currently being discovered using metagenomics and metataxonomics as primary methodologies. Metataxonomics, or amplicon sequencing (AS), focuses on specific genomic regions such as 16S and 18S ribosomal DNA (rDNA) to create taxonomic profiles limited to a single domain of microorganisms. AS can only characterize microbes down to the genus level, whereas metagenomics, or whole genome shotgun sequencing (WGS), provides a broader approach to characterize functional and taxonomic profiles across all microbial domains, down to the species or strain level. WGS can excel in detecting lower abundance microbes compared to AS but requires a higher cost and more complex bioinformatic analysis [29,30,31]. Furthermore, the fact that 98% of bacteria are unculturable suggests that these methodologies are powerful for uncovering previously hidden microbial diversity, despite their limitations and resource demands [32,33].

The current study aims to utilize WGS to uncover distinctive features of bacteria and fungi in the rhizosphere microbiomes associated with the plant *M. crassifolia*, as well as those present in neighboring bulk soil microbiomes.

## 2. Materials and Methods

### 2.1. Samples Collection

**Figure 1:**
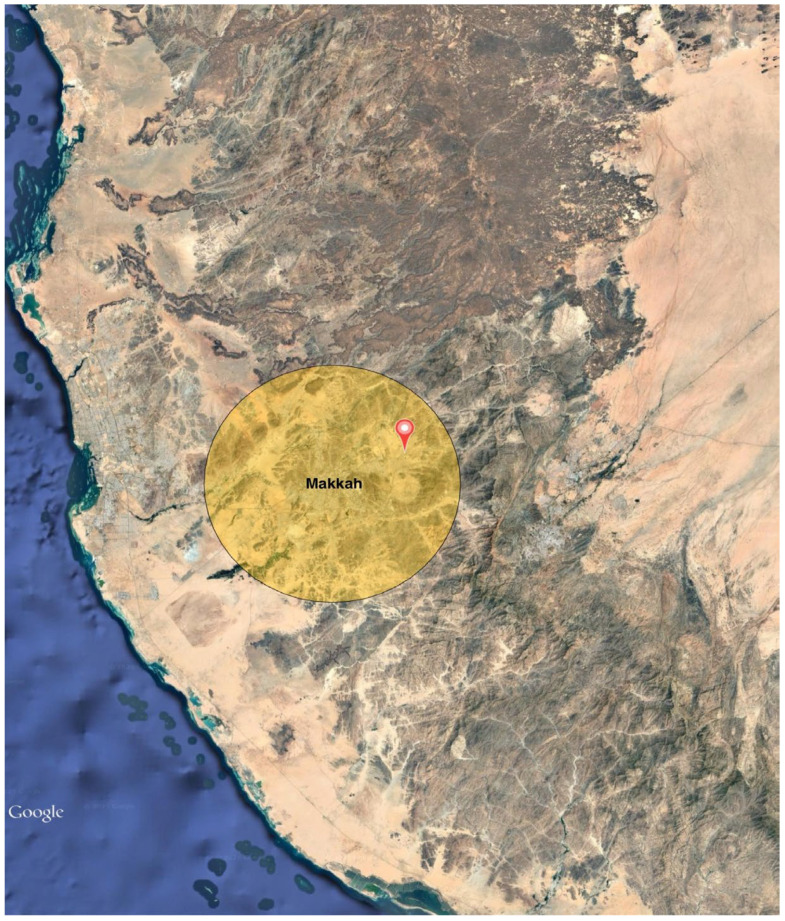
The figure depicts the location where the samples were collected.

The soil was sampled in the morning during October 2023 from the *M. crassifolia* plant located in the Makkah district of southeastern Saudi Arabia, at coordinates 21°30′23.8″ N latitude and 40°01′35.1″ E longitude at an altitude of 439 m above sea level (Figure 1). Eight samples, each weighing 5 g, were collected, four of them targeting the upper 0–10 cm layer of bulk soil and four from the rhizosphere at depths between 18 and 25 cm [34,35]. Immediately after collection, the soil samples were rapidly frozen in liquid nitrogen and subsequently stored on dry ice, then transferred at −20 °C until later use. A soil–water slurry was prepared at a 1:1 dry-weight ratio to measure the soil’s pH.

### 2.2. DNA Extraction

DNA extraction was conducted using the PowerMax^®^ Soil DNA Isolation Kit (MO BIO Laboratories, Carlsbad, CA, USA) following the guidelines provided by the manufacturer. Next, the DNA integrity was assessed through electrophoresis on a 0.8% agarose gel, while the purity of the DNA was determined using a NanoDrop Spectrophotometer (Thermo Fisher Scientific, Waltham, MA, USA) as a pre-initial step in the Quality Control (QC) procedures.

### 2.3. Library Preparation and DNA Sequencing

Library preparation and DNA sequencing tasks were conducted at Novogene Co., Ltd. in Singapore. All procedures utilize one microgram of sample DNA as input. The initial QC measures included quantification, integrity assessment and purity analysis of the DNA samples using the Agilent 5400 system (Santa Clara, CA, USA). Following this step, DNA fragmentation was achieved via sonication. DNA fragments then underwent end-polished, phosphorylation and A-tailing reactions. Subsequently, the fragments were ligated with Illumina adapters and amplified using polymerase chain reaction (PCR). Library preparation was conducted using the Illumina Ultra DNA Library Prep kit (NEB, Ipswich, MA, USA), followed by PCR primer removal. Size selection was performed to isolate DNA fragments of 350 base pairs (bp). Library quantification was conducted using Qubit and real-time PCR. Library size distribution was assessed using a bioanalyzer as a secondary QC measure. Finally, the pooled libraries were sequenced on the Illumina Novaseq^TM^ platform 6000 (San Diego, CA, USA).

### 2.4. Bioinformatics Analysis

Clean data reads extraction was performed using Readfq software (https://github.com/cjfields/readfq, 20 January 2025). Reads were subjected to a stringent QC process involving three stages. First, reads exceeding a 10 bp threshold with N bases were removed. Second, reads that had adapter overlaps greater than 15 bp were discarded. Third, reads that contained a proportion of 40 bp of low-quality bases (Phred score ≤ 38) were filtered out. Then, the reads were aligned to the plant reference genome of *M. crassifolia* using Bowtie2 software (version 2.2.4) to identify and eliminate any residual plant-derived sequences [36,37,38]. Genome assembly was conducted using MEGAHIT software (version 1.0.4) [37,39,40]. To capture genomic information from low-abundance microbial taxa potentially underrepresented in the initial assembly, all unused scaffolds were combined into a composite sample designated NOVO_MIX. This composite sample was then subjected to an independent de novo assembly. All scaffolds containing “N” base regions were excluded from the final scaffold following assembly. This fourth QC step involved fragmentation of the scaffolds at the site of each “N” base. Finally, the clean sequencing data were aligned to the reference genome using Soap software version 2.21 to generate usable scaftig sequences [39,41].

Open reading frames (ORFs) indicative of potential genes were identified within scaftig sequences ≥ 500 bp in length using MetaGeneMark software version 2.10 [36,42,43,44,45]. Identified ORFs shorter than 100 nucleotides were excluded [40,41,46,47,48]. To establish a non-redundant catalog of identified genes from the assembled metagenome, redundant sequences with coverage of ≥90% and a sequence identity of ≥95% were eliminated using the Cluster Database at High Identity with Tolerance (CD-HIT) software (version 4.5.8) [42,45,47,49,50]. The abundance of genes in the non-redundant genes catalog (nrGC) was then determined. A computational pipeline developed by Novogene Corporation facilitated the categorization of these data at the gene level. As a fifth QC measure, longer metagenomic segments, termed “contigs”, were subjected to taxonomic classification that depended on consistent annotation. Contigs were classified as putatively hybrid, and thus erroneous or chimeric, if less than 95% of their constituent reads could be assigned to a single reported species. This singularity threshold allowed for identifying and excluding contigs with ambiguous taxonomic classification. The clean sequencing data were then aligned to the nrGC using Bowtie2 software. This alignment enabled the quantification of mapped reads for each unique gene, providing a measure of the relative abundance (RA) of each gene within the samples. Genes with ≤2 mapped reads, indicating very low abundance, were removed [41,47]. The remaining gene set constituted the final unigene catalog. Gene abundance within this final catalog was quantified based on the length of the corresponding gene sequence and the number of mapped reads [51,52,53]. Several downstream bioinformatic analyses were performed. These analyses included the generation of basic descriptive statistics, core–pan gene analyses and Venn diagram visualizations.

Unigene sequences were compared to the National Center for Biotechnology Information (NCBI) non-redundant (NR) protein database using DIAMOND software (version 2.1.8), which implements a BLASTP (NCBI) search algorithm [37,51]. The taxonomic annotation was determined using the Lowest Common Ancestor (LCA) method to account for sequences with multiple alignments. The LCA algorithm traverses the NCBI taxonomic tree for each alignment to identify the most specific common ancestor taxon. This annotation process was performed using MEGAN software (version 4). Gene abundance tables were generated by combining sequence read counts with LCA annotation data at different taxonomic levels. Taxon abundance within the sample was determined based on the cumulative read counts for all genes associated with that taxon [36,42,54,55]. The visualization of data was conducted utilizing the Matplotlib package (version 3.10.0) within a Python environment (version 3.11.11.).

### 2.5. Statistical Analysis

Beta diversity was calculated to examine taxonomic composition based on RA at different taxonomic levels. General trends in Beta diversity and clustering of samples within the studied groups were assessed by principal component analysis (PCA) of the taxonomic abundance data using the ade4 package in the R statistical computing environment. To statistically evaluate significant differences in microbial community profiles between groups relative to within groups, an analysis of similarities (ANOSIM) was performed using the vegan package in R statistical computing environment. In addition, to identify specific taxa that showed significant differential abundance between the designated sample groups, a differential abundance analysis was conducted using the Linear Discriminant Analysis (LDA) Effect Size (LEfSe) with a default LDA threshold of 4 [56].

## 3. Results

WGS was utilized to analyze the microbiome of the rhizosphere and bulk soil of *M. crassifolia* plants, allowing the detection of bacterial and fungal structures in each soil type. The soil samples were collected at a temperature of 34 °C and a pH of 7.6. According to climatological data for the Makkah region from the Climate Change Knowledge Portal, the average annual rainfall is 95.71 mm. A previous study in this region reported soil organic matter (SOM) concentrations ranging from 0.01 to 4.5 mg kg^−1^, with higher levels observed in the southern areas, including the present study area [2]. The microbiome groups were categorized according to soil type: group ’A’ represents the rhizosphere microbiome, while group ’B’ represents the bulk soil microbiome. In addition, ’R’ stands for individual samples from the rhizosphere, and ’S’ for individual samples from the bulk soil. Detailed statistics on raw metagenomic data can be found in Table 1 and clean data in Table S1, while a Venn diagram is shown in Figure S1; both are available in the Supplementary Document. The distribution of ORF lengths (measured in nucleotides (nt)) and scaffold lengths (for sequences of 500 bp or longer, also known as scaftigs) are displayed in Table S2. Core–pan rarefaction curves, which depict the identification of shared and distinct ORFs in the metagenomic datasets as more samples are included, are provided in Figure S2.

### 3.1. Analysis of Similarities (ANOSIM)

**Figure 2:**
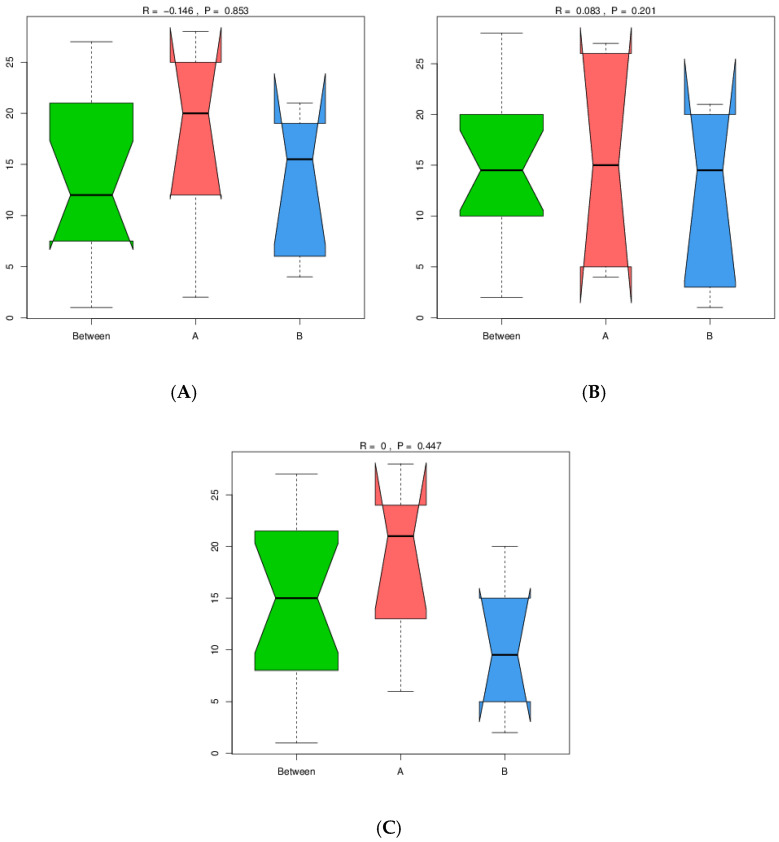
ANOSIM results of soil metagenomes associated with *M. crassifolia* plants. ANOSIM was used to compare microbial community structure between groups A and B at the (**A**) phylum, (**B**) genus and (**C**) species levels.

ANOSIM was employed to assess compositional differences in microbial communities between groups A and B. While the ANOSIM analysis revealed slight differences between groups A and B at the phylum level (R = −0.146), this difference was not statistically significant (*p* = 0.853). A similar result was observed at the genus level. At the species level, the ANOSIM test indicated no significant difference in community composition between the two groups (R = 0, *p* = 0.447) (Figure 2A–C).

### 3.2. Principal Component Analyses (PCA)

**Figure 3:**
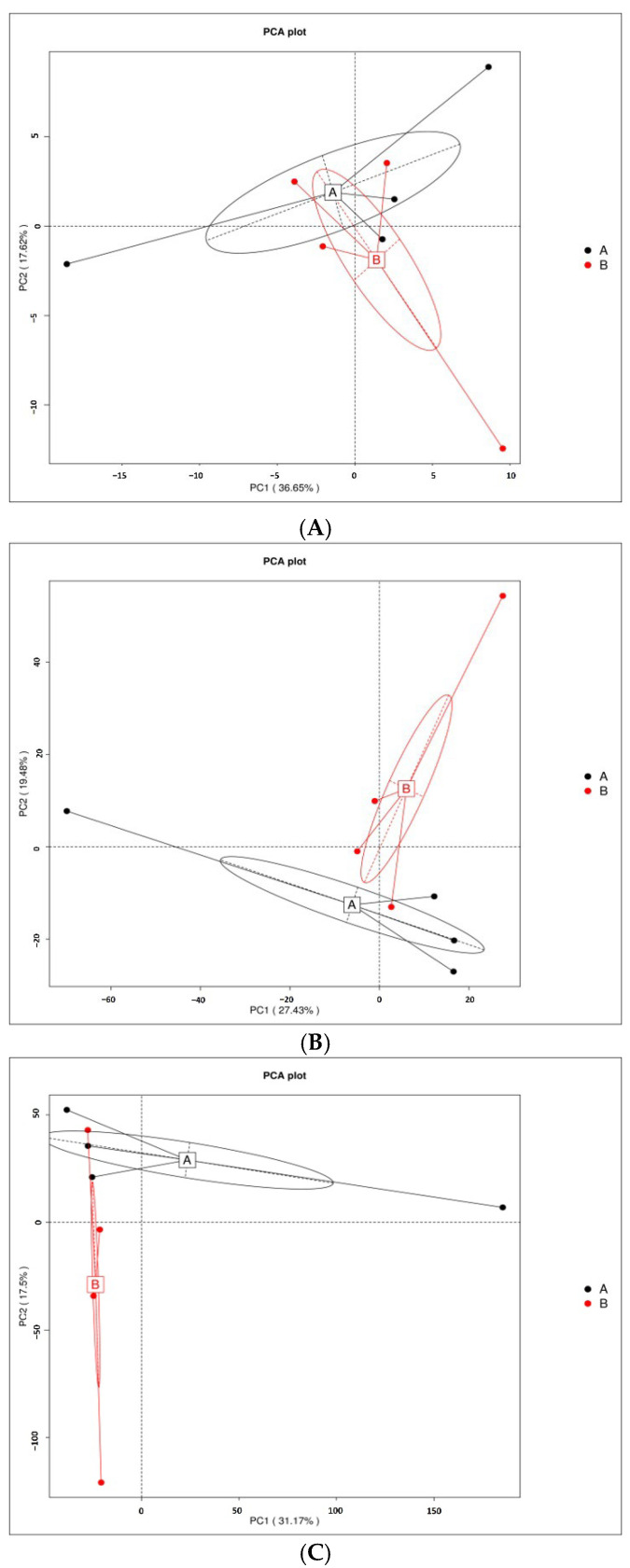
PCA results of soil metagenomes associated with *M. crassifolia* plants. PCA was performed based on gene abundances at the (**A**) phylum, (**B**) genus and (**C**) species levels. The *x*-axis and *y*-axis represent the first and second principal components (PC1 and PC2), respectively. The percentage of variation explained by each principal component is indicated in parentheses.

PCA revealed differences in microbiome composition between rhizosphere and bulk soil samples at the phylum, genus and species levels. At the phylum level, rhizosphere microbiomes clustered primarily along the positive side of the PC2 axis, while bulk soil microbiomes were positioned primarily along the positive side of the PC1 axis. At the genus level, rhizosphere microbiomes clustered toward the negative side of the PC1 axis, while bulk soil microbiomes clustered toward the positive side of the PC2 axis. A similar trend was observed at the species level, with rhizosphere microbiomes clustering toward the negative side of the PC1 axis, and bulk soil microbiomes exhibiting the opposite pattern. The distinct clustering of rhizosphere and bulk soil samples across the phylum, genus and species levels indicates significant differences in microbial composition between soil types (Figure 3A–C).

### 3.3. Microbial Annotation

**Figure 4:**
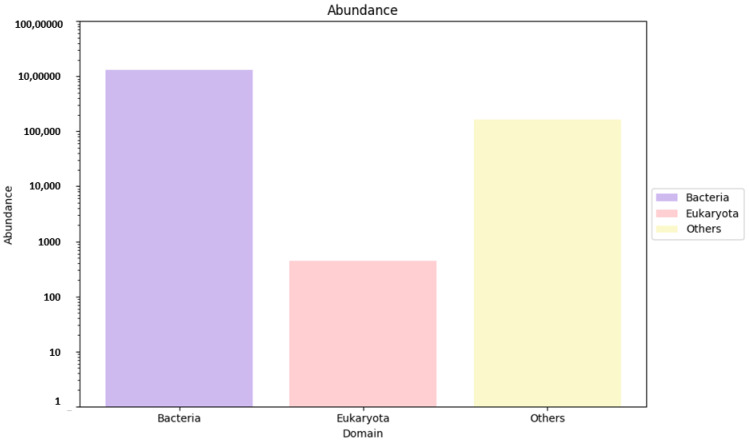
Microbial abundance at the domain level, representing bacteria, Eukaryota and others. The abundance values are based on nRG identified in metagenomic data of *M. crassifolia* plant across soil types. “Others” include archaea, viruses and unidentified taxa.

As mentioned above, gene predictions and mapping were performed for the assembled scaftigs (Table S2) and ORFs were identified (Table S2). Non-redundant genes (nRGs) were then clustered, leading to the development of catalogs of the original nrGCs. Gene abundances were then classified at three different taxonomic levels, including phylum, genus and species. This was followed by an analysis of RA. Archaea and viruses were excluded and are shown as ’other’ with an unidentified taxon. However, the annotation indicates that detailed information is not available for most taxa in the metagenomes, as evidenced by the high gene abundance of the unknown taxa. Furthermore, much of this unidentified genetic information corresponds to the high proportion of low-abundance, unculturable microorganisms. The analysis revealed a significantly higher abundance of bacteria compared to eukaryotes (domain that encompasses fungi) (Figure 4). The top ten most predominant taxa across soil types (e.g., rhizosphere and bulk soil) were selected for further analysis at the three taxonomic levels (phylum, genus and species).

**Figure 5:**
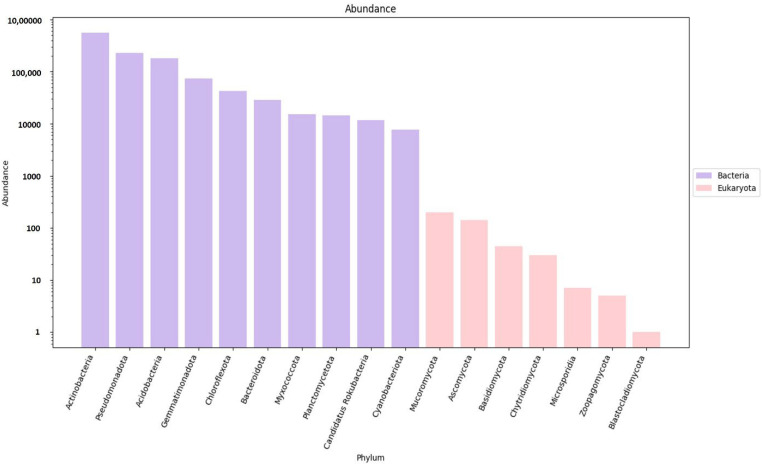
The microbial abundance of the top ten bacterial (purple columns) and eukaryotic (pink columns) phyla, based on nRG identified in the metagenomic data of the *M. crassifolia* plant across different soil types, including the NOVO_MIX samples.

**Figure 6:**
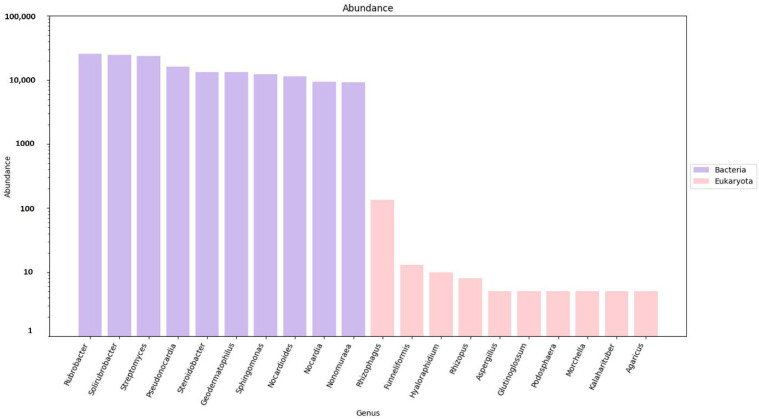
The microbial abundance of the top ten bacterial (purple columns) and eukaryotic (pink columns) genera, based on nRG identified in the metagenomic data of the *M. crassifolia* plant across different soil types, including the NOVO_MIX samples.

**Figure 7:**
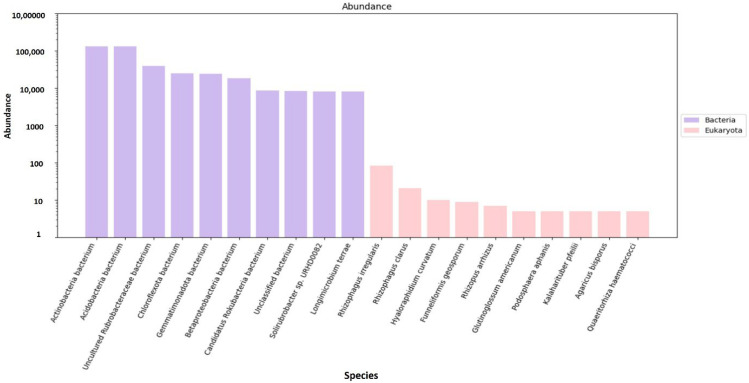
The microbial abundance of the top ten bacterial (purple columns) and eukaryotic (pink columns) species, based on nRG identified in the metagenomic data of the *M. crassifolia* plant across different soil types, including the NOVO_MIX samples.

**Figure 8:**
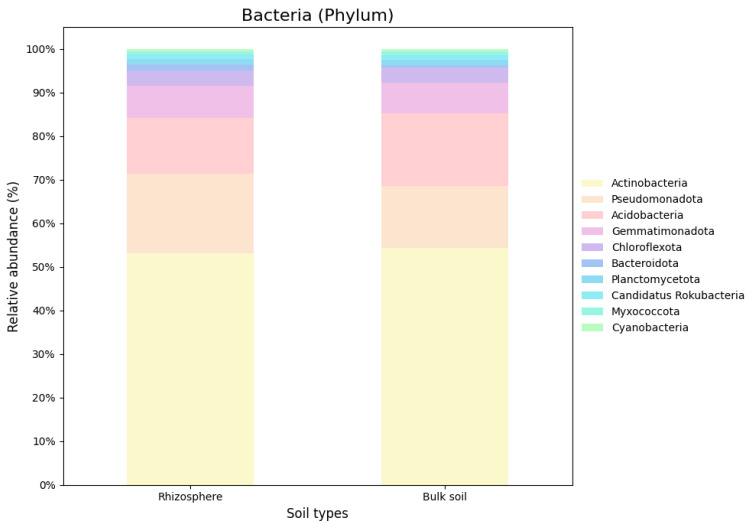
The relative microbial abundance of the top ten bacterial phyla, based on nRG identified in the metagenomic data of the *M. crassifolia* plant across different soil types (e.g., rhizosphere and bulk soil).

**Figure 9:**
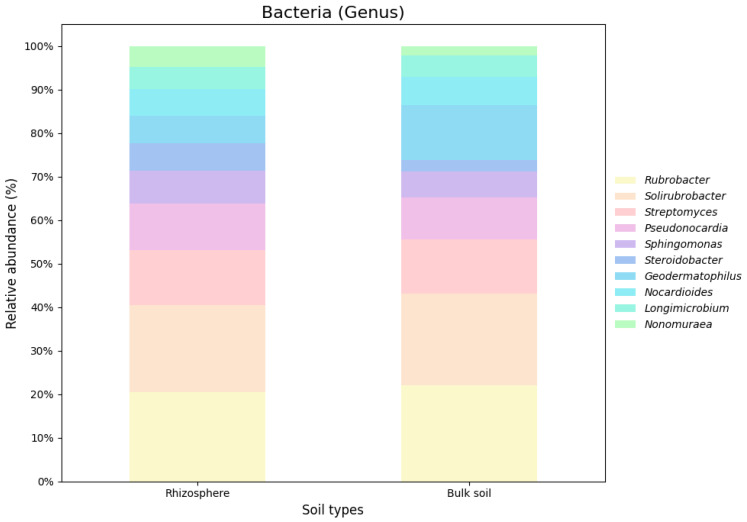
The relative microbial abundance of the top ten bacterial genera, based on nRG identified in the metagenomic data of the *M. crassifolia* plant across different soil types (e.g., rhizosphere and bulk soil).

**Figure 10:**
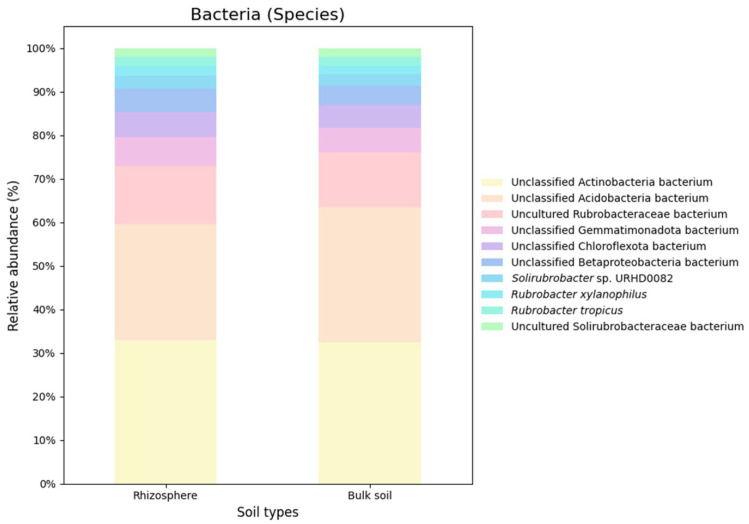
The relative microbial abundance of the top ten bacterial species, based on nRG identified in the metagenomic data of the *M. crassifolia* plant across different soil types (e.g., rhizosphere and bulk soil).

**Figure 11:**
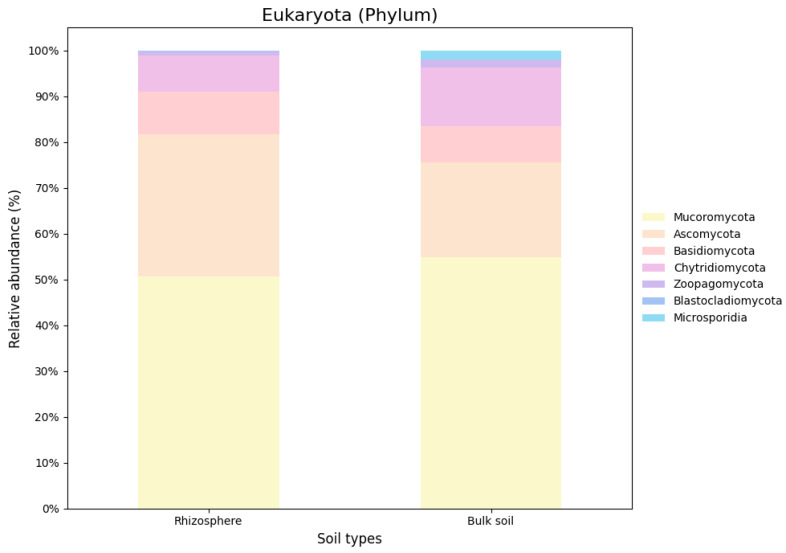
The relative microbial abundance of the top ten eukaryotic phyla, based on nRG identified in the metagenomic data of the *M. crassifolia* plant across different soil types (e.g., rhizosphere and bulk soil).

**Figure 12:**
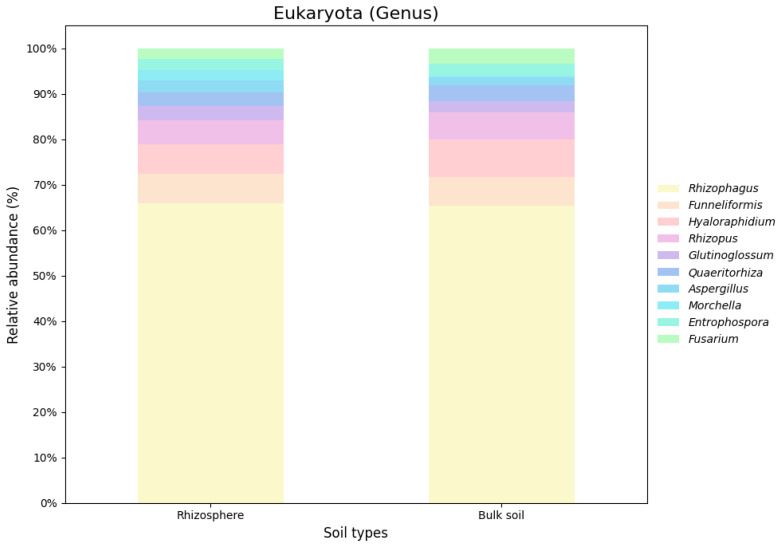
The relative microbial abundance of the top ten eukaryotic genera, based on nRG identified in the metagenomic data of the *M. crassifolia* plant across different soil types (e.g., rhizosphere and bulk soil).

**Figure 13:**
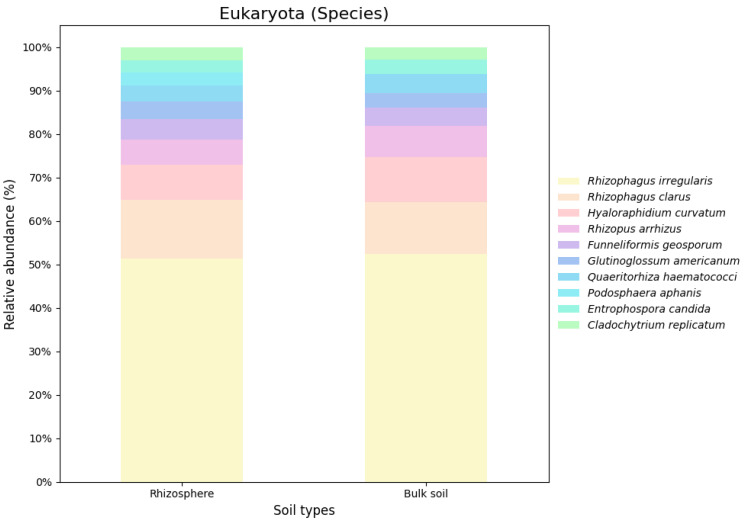
The relative microbial abundance of the top ten eukaryotic species, based on nRG identified in the metagenomic data of the *M. crassifolia* plant across different soil types (e.g., rhizosphere and bulk soil).

Based on the nRG, the phyla Actinobacteria, Pseudomonadota and Acidobacteria were identified as the most prevalent among bacteria, while Mucoromycota, Ascomycota and Basidiomycota were the most prevalent among fungi (Figure 5). At the genus level *Rubrobacter*, *Solirubrobacter* and *Streptomyces* were revealed as the dominant bacterial taxa, while *Rhizophagus*, *Funneliformis* and *Hyaloraphidium* were the most abundant fungal genera (Figure 6). At the species level, unclassified members of the Actinobacteria and Acidobacteria, along with an uncultured Rubrobacteraceae bacterium, were the most abundant bacterial taxa. Within the fungi, *Rhizophagus irregularis*, *Rhizophagus clarus* and *Hyaloraphidium curvatum* were the dominant species (Figure 7). Figure S3 shows a comparative analysis of the samples without NOVO_MIX. To investigate the RA of microbial taxa in different soil types (e.g., rhizosphere and bulk soil), the taxonomic assignment of nRGs in metagenomes was analyzed. This analysis was performed at the phylum, genus and species levels for bacteria (Figure 8, Figure 9 and Figure 10 and Figures S4–S6) and Eukaryota (Figure 11, Figure 12 and Figure 13 and Figures S7–S9). As some of the taxa were not detected in the soil type-specific analyses, their occurrence is attributed to the NOVO_MIX group. To allow for contrasts in the RA of fungi and bacteria between the rhizosphere and bulk soil, an initial analysis was performed to assess the overall abundance of microorganisms in the combined dataset that includes the NOVO_MIX group. The NOVO_MIX category consists of short gene sequences collected from all samples; therefore, its abundance cannot be assigned to a specific soil type. The present approach establishes a basic understanding of the microbial community composition before investigating specific variations between the rhizosphere and bulk soil. Although slight differences in RA between soil types were detected, the next discussion will focus on relative rather than absolute abundance.

### 3.4. Linear Discriminant Analysis (LDA) Effect Size (LEfSe)

**Figure 14:**
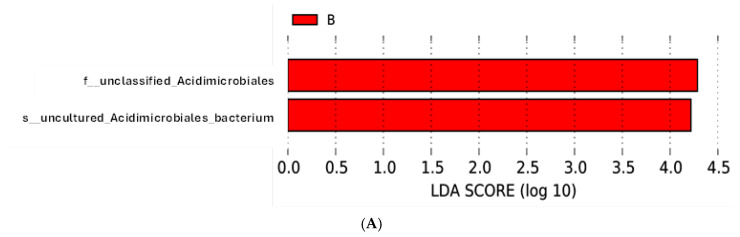

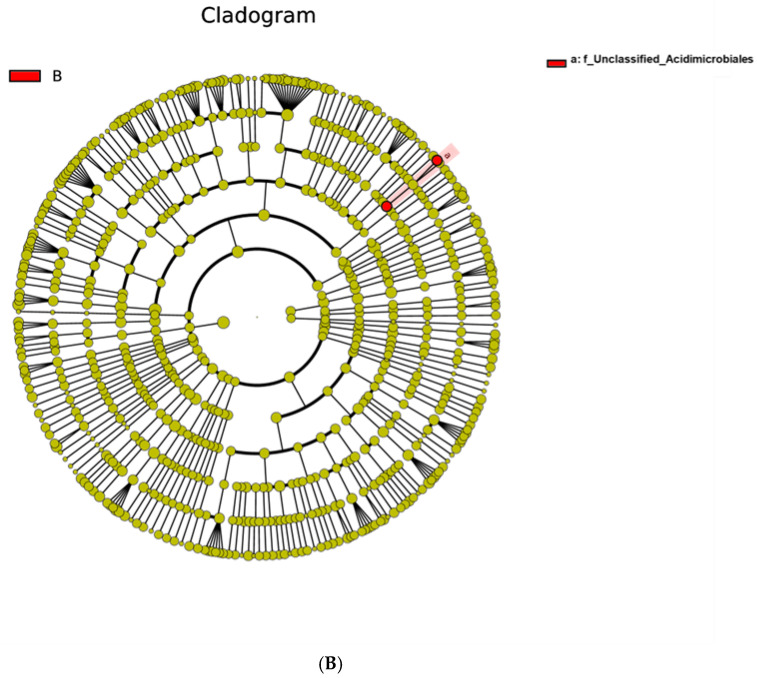
(**A**) The LEFSe line graph represents species that show a significant difference in abundance, with an LDA threshold of 4.0. The length of each bar in the graph is proportional to the size of the effect (LDA score), with longer bars indicating greater differences in abundance. The *x*-axis represents the LDA score for the bulk soil ‘B’ group. (**B**) The phylogenetic tree illustrates the relative abundance and evolutionary links between taxa. The nodes on the tree denote taxonomic classifications, while the branching patterns indicate putative ancestral relationships. The size of each node correlates with the observed abundance of that taxon. The highlighted red node labeled “a” identifies “Unclassified Acidimicrobiales” as a biomarker taxon distinct from the indistinguishable yellow nodes. The red marker “B” means that this biomarker taxon is associated with the bulk soil habitat.

LEfSe software (version 1.1.2.) was employed to identify biomarkers exhibiting significant variations in abundance between A and B groups, enabling the detection of differential species abundance between groups A and B. Within the bulk soil microbiome, one taxonomic biomarker exhibited statistically significant enrichment (*p* ≤ 0.05, LDA score (log<sub>10</sub>) > 4.0). This biomarker, identified at family and species levels, corresponds to an uncultured bacterium within the order Acidimicrobiales (phylum Acidobacteria) (Figure 14A,B).

## 4. Discussion

Fungi are ecologically important, but their diversity, especially in the dry and semi-arid Arabian Peninsula, has been little investigated compared to bacteria [57]. This work uses metagenomics WGS to define the top 10 bacterial and fungal structures of M. *crassifolia’s* rhizosphere and bulk soil at the phylum, genus and species levels. Due to its superior taxonomic resolution, WGS may find bacterial and fungal species SA missed [30,58]. This precision is significant because soil microbial populations are susceptible to complex biotic and abiotic variables like drought [59,60]. AS is useful for processing large datasets cheaply and efficiently, but it fails to capture microbial community diversity. Soil environments’ great microbial diversity and intricate interactions show these limits. Since most 16S rDNA primers do not bind to Planctomycetota’s sequences, PCR bias makes identification problematic [61,62]. AS-based research is sometimes called “metagenomics” instead of “metataxonomics.” This distinction is important for scientific clarity since metagenomics analyzes genetic material from whole microbial communities, whereas metataxonomics explore taxonomic diversity, reflecting AS-based research priorities. Mislabeling “metagenomics” confounds academics and concepts. Clear communication that achieves research aims requires technique-appropriate terminology.

Microorganisms are considered PGPMs based on a set of specific criteria. It must fulfill at least two of the following three requirements: enhancement of plant development, successful colonization and mitigation of biotic and abiotic stressors [63]. It is important to note that the efficiency of plant growth promotion is not solely determined by the quantity of plant growth-promoting (PGP) activities. Rather, each strain’s unique combination and mechanisms play an essential role in its effectiveness. Therefore, to optimize plant development, it is necessary to identify and analyze the most suitable PGP properties for each specific application [64].

The current investigation found the ten most prevalent bacterial phyla in *M. crassifolia* rhizosphere and bulk soil. The core phylum in the rhizosphere and bulk soils was actinobacteria. Significant compositional variations were found across soil types. Pseudomonadota and Bacteroidota predominated in the rhizosphere. Acidobacteria was more abundant in bulk soil. Gemmatimonadota, Chloroflexota, Candidatus Rokubacteria, Myxococcota, Planctomycetota and Cyanobacteria were consistent throughout both soil types (Figure 8). *Pseudonocaradia*, *Sphingomonas*, *Steroidobacter* and *Nonomuraea* had increased rhizosphere abundances. In contrast, bulk soil *Rubrobacter*, *Solirubrobacter* and *Geodermatophil* were abundant. The two soil types had similar abundances of *Streptomyces*, *Nocardioides* and *Longimicrobium* (Figure 9). At the species level, unclassified Actinobacteria bacterium had a higher RA in the rhizosphere than in the bulk soil, whereas unclassified Acidobacteria had a higher prevalence in the bulk soil. Both environments had comparable RA for the remaining species (Figure 10). Current findings match Actinobacteria abundance in drought-affected soils [65]. Actinobacteria regulate ethylene levels, lowering plant stress [66]. During drought, plants generate ethylene, but too much might limit growth [67]. *Streptomyces* spp., for example, produces antibiotics and other secondary metabolites [68,69,70,71,72,73]. Rhizosphere and bulk soil have greater *Streptomyces* spp. RA. *Nocardioides* spp. dominated both soil types, showing their widespread prevalence. The genus produces phytohormones including salicylic acid (SA) and indole-3-acetic acid. SA helps plants develop and IAA helps them deal with salt [74,75]. *Pseudonocardia* and *Nonomuraea* predominated in the rhizosphere, unlike the bulk soil. IAA production makes *Nonomuraea* and *Pseudonocardia* spp. suitable PGPMs. *Nonomuraea* is antifungal and *Pseudonocardia* species is antimalarial [76,77,78,79]. Due to intense colonization by several taxa, *Geodermatophilus* spp. is abundant in bulk soil.

Acidobacteria were more abundant in bulk soil, while Pseudomonadota and Bacteroidota dominated the rhizosphere, due to their differing nutritional needs. The oligotrophic nature of Acidobacteria, favoring low-nutrient environments, aligns with their prevalence in the nutrient-poor bulk soil. In contrast, copiotrophic Pseudomonadota and Bacteroidota thrive in carbon-rich conditions, consistent with their dominance in the rhizosphere, enriched by root exudates. Root exudates, through chemotaxis, may actively recruit PGPMs from the bulk soil, functioning as a reservoir of potential symbionts [80]. On the one hand, Pseudomonadota acts as a PGPM by fixing nitrogen, producing IAA and solubilizing phosphorus, which is especially beneficial during droughts [81,82,83,84]. Bacteroidota is known for its ability to produce the enzyme 1-aminocyclopropane-1-carboxylate (ACC) deaminase [85]. Studies have associated soil degradation and reduced fertility with a decline in Bacteroidota abundance, making it a potential indicator of soil quality [86]. On the other hand, Acidobacteria members, though less prevalent in the rhizosphere than in bulk soil, exhibit root colonization, potentially facilitated by their production of exopolysaccharide (EPS). The presence of Acidobacteria species suggests a symbiotic relationship with plants, which could precede their function as PGPMs. This symbiotic interaction contributes to plant growth promotion by regulating key metabolic processes (sulfur, nitrogen and carbon cycling) and improving soil health via EPS-mediated soil structure enhancement. It also enhances plant growth directly through IAA production and indirectly by increasing iron availability via siderophore production [87,88].

Higher soil macronutrients, plant species richness and Gemmatimonadota abundance are positively correlated [89]. This investigation found a hitherto uncharacterized microbial species from this phylum, but its restricted culturability prevents a full elucidation of its functional significance in plant–microbe interactions [90]. It is premature to categorize Gemmatimonadota as a PGPM. Candidatus Rokubacteria is uncultivable, preventing functional characterization. The phyla Planctomycetota and Myxococcota cause *Panax notoginseng* root rot [91], although Myxococcota also respires, denitrifies and ammoniifies [92]. Some Myxococcota species modulate rhizosphere microbiome structure as Fusarium wilt biocontrol agents [93]. This apparent contrast reflects the complex dynamics of plant–microbe systems, where plant species and root exudate components affect how particular bacterial taxa affect plant health [94].

Some reports indicate a strong link between Chloroflexi and the rhizosphere of medicinal plants, as in the present study [95]. The current results also confirm the presence of Cyanobacteria in the rhizobiome of arid regions in western Saudi Arabia, aligning with previous studies [96]. Cyanobacteria, known for their potential as biofertilizers [97,98,99,100,101,102], enhance plant growth through nitrogen fixation, phytohormone production, phosphate solubilization and pathogen control [98,102,103,104]. Their production of auxins, cytokinin, SA and gibberellic acid is critical in alleviating abiotic stress and promoting plant development, especially during drought [98,103,105,106,107,108,109,110].

Previous studies have shown that *Sphingomonas* spp. act as PGPMs by mitigating abiotic stress and improving nutrient uptake [111,112,113,114]. *Solirubrobacter* and *Geodermatophilus* spp. contribute to nutrient cycling, with the former exhibiting resistance to UV radiation, drought and carbon limitation [84,111,112,113,114,115,116]. *Rubrobacter* spp. possess photosynthetic proteins and are involved in the carbon, nitrogen and sulfur cycles [117,118,119]. *Steroidobacter* spp. confer cadmium resistance [120], while Betaproteobacteria species play a role in nitrogen fixation, degradation of aromatic compounds, inhibition of pathogens and arsenic resistance [121,122,123,124]. *Longimicrobium* spp. improve heavy metal resistance and plant biomass [125].

The present study also revealed a significant prevalence of unidentified bacterial species in all soil types, especially in the phyla Actinobacteria, Gemmatimonadota, Chloroflexota and Betaproteobacteria, suggesting that they could serve as new candidates for PGPMs. In the bulk soil, an unclassified Acidobacteria was identified as the most dominant species after Actinobacteria. These unclassified bacteria may indirectly have the potential to promote plant growth via soil as novel taxa, emphasizing the need for further investigation.

The top ten phyla observed in the rhizosphere and bulk soil microbiomes at the Eukaryota domain shared dominance, although their relative abundances varied. Notably, Mucoromycota was present in both soil types but showed higher levels in the bulk soil. On the other hand, Ascomycota exhibited greater abundance in the rhizosphere while Chytridiomycota was more prevalent in the bulk soil. Basidiomycota and Zoopagomycota displayed similar abundance levels in both regions, whereas Blastocladiomycota were only found in the rhizosphere. The RA of genera and species was similar across both regions, with a few notable exceptions. *Podosphaera aphanis* was exclusively detected in the rhizosphere, and *Morchella* spp. and *R. clarus* exhibited higher RA in the rhizosphere. In contrast, *H. curvatum* and *Rhizopus arrhizus* demonstrated greater RA in the bulk soil (Figure 11, Figure 12 and Figure 13).

Zygomycetous fungi (Mucoromycota and Zoopagomycota), which lack zoospores, have important ecological roles. Their influence is attributed to plankton and pathogen population management, nutrient cycling within food webs and heavy metal bioremediation [126]. Despite resource limitations, Ascomycota significantly contribute to carbon and nitrogen cycling through decomposition, symbiotic relationships and pathogenic interactions. Their enzymatic arsenal (cellulases, hemicellulases and ligninases) and effector proteins mediate these interactions with plants [127]. Basidiomycetes, prevalent in warm and humid climates, are noteworthy for producing bioactive secondary metabolites with antioxidant and anti-pathogenic properties [128]. Zoosporic fungi, including Chytridiomycota, demonstrate ecological versatility as saprotrophs and parasites, employing diverse enzymatic mechanisms [129]. Likewise, the lately delineated Blastocladiomycota phylum includes notable plant pathogens such as *Physoderma* spp. [130,131]. Microsporidia, found in diverse soil environments, including tomato rhizosphere, often serve as biocontrol agents or act as pathogens targeting insect species [132,133].

Symbiotic root colonization and nutrient exchange by AMFs including *R. clarus*, *R. irregularis* and *Funneliformis geosporum* increase host plant drought tolerance [134]. However, dryness significantly limits AMF development and activity, reducing the efficacy of this symbiotic interaction in relieving water stress [135]. The lack of phytase-encoding genes in AMF species limits their ability to metabolize organic phosphorus, requiring cooperative interactions with phosphate-solubilizing bacteria (PSB). Fungal exudates induce PSB migration toward AMF hyphae, which activates PSB chemotaxis genes. The chemotactic recruitment of PSB is necessary for this symbiotic phosphorus acquisition approach to work [136]. Several fungal species improve plant stress tolerance. *R. clarus* improves soybean and tomato drought tolerance, boosting growth and recovery [137,138,139]. *R. irregularis* improves root architecture, glomalin production and microbial communities in different plants to reduce water stress. These effects improve nutrient absorption, secondary metabolites and antioxidants [140,141,142,143,144,145,146]. *F. geosporum*, found in *Capsicum annuum*, *Celtis caucasica* and *Zingiber* spp. rhizospheres, promotes *Solanum tuberosum*, *C. annuum* and *C. caucasica* growth and reduces legume salinity stress [147,148,149,150,151]. By enhancing phosphorus uptake, *Entrophospora candida* boosts *Allium vineale* development [152].

**Figure 15:**
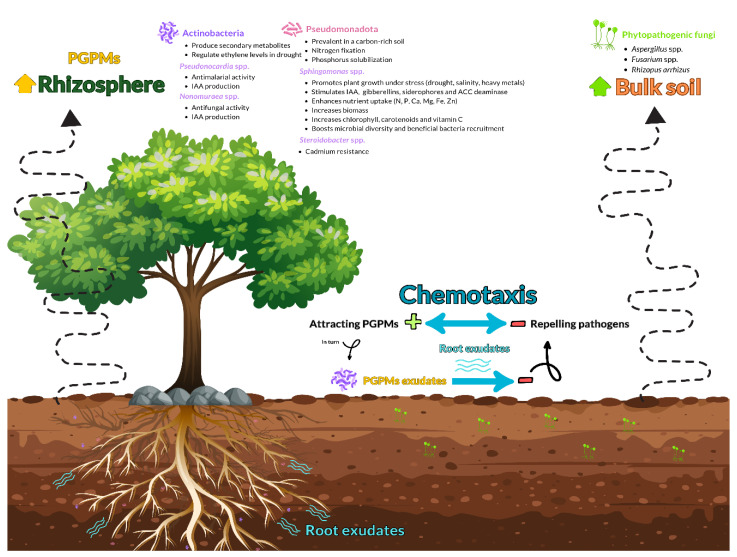
The microbiome of the rhizosphere is mainly structured by chemotaxis, i.e., the controlled movement of microbes in response to chemicals. This behavior can be described as either negative chemotaxis, in which microbes move away from a chemical repellent, or positive chemotaxis, in which they move toward a chemical attractant. Under the guidance of chemical cues from root exudates, PGPMs show positive chemotaxis towards plant roots. These microbes can colonize the rhizosphere more easily thanks to this controlled migration, which improves plant development. On the other hand, certain chemicals contained in root exudates or released by PGPMs, such as *Nonomuraea* spp. in the rhizosphere, can induce negative chemotaxis on phytopathogens, including *Aspergillus* spp., *Fusarium* spp. and *R. arrhizus*. This repellent effect restricts their access to the rhizosphere, confining them to the bulk soil and protecting the plant.

On the contrary, the presence of the strawberry powdery mildew pathogen *P. aphanis* in the rhizosphere is likely non-specific, resulting from wind or sand dispersal [153]. The plant pathogenic fungi, such as *R. arrhizus*, *Aspergillus* spp. and *Fusarium* spp., detected in bulk soil, may be passively displaced from rhizospheres via chemotaxis mediated by bacteria or root exudates [154,155,156] (Figure 15). Intriguingly, some of these pathogens may indirectly benefit plants by catabolizing complex organic molecules into simpler, more readily utilizable forms [157,158].

The presence of Actinobacteria, particularly *Streptomyces*, in soil has been correlated with the occurrence of Ascomycota, Basidiomycota, Mucoromycota, Pseudomonadota and Bacteroidota in the rhizosphere of *Ziziphus jujuba* [159]. This suggests a positive interaction between Actinobacteria and these fungal and bacterial phyla, in agreement with present findings. However, it is essential to recognize that multiple factors beyond microbial interactions influence such associations in the rhizosphere. Plant species, root exudates, soil pH, temperature, moisture levels and organic matter content shape these relationships [160]. Thus, it cannot be definitively concluded that these associations are for the rhizosphere of all plant species. Furthermore, the present study observed the presence of Chytridiomycota and Cyanobacteria, which has been corroborated by prior research [161]. Plant–microbe signaling networks mediate the structuring of rhizosphere bacterial communities, resulting in differential distribution of certain taxa compared to bulk soil. However, drought exerts a stronger influence on bacterial community composition than plant species identity. On the other hand, fungal community composition is primarily driven by soil properties, reflecting the saprophytic nature of many fungi and their reliance on SOM decomposition rather than plant-derived carbon only [162,163,164,165].

## 5. Conclusions

The present study illustrates the bacterial and fungal communities inhabiting the *M. crassifolia* rhizosphere and surrounding bulk soil using WGS. Our results indicate intricate symbiotic relationships within the rhizosphere microbiome, including associations between AMF and PSB. Additionally, the analysis of the results suggests that specific microbial taxa may enhance plant resilience to biotic and abiotic stresses. Significantly, this study also identified novel microbial taxa not currently represented in existing databases, highlighting the unexplored diversity within the *M. crassifolia* rhizosphere. These unclassified microbes may represent a promising avenue for future research, with the potential to uncover novel PGPMs. To advance research on *M. crassifolia* and its soil rhizosphere microbiome, future studies should focus on isolating and characterizing microbial taxa within the Actinobacteria, Betaproteobacteria, Gemmatimonadota and Chloroflexota phyla to uncover novel plant growth-promoting microbes and their roles in nutrient cycling and plant resilience in arid conditions. Additionally, the molecular mechanisms underlying the tripartite interactions between AMF, PSB and *M. crassifolia* plant should be explored, with an emphasis on nutrient uptake and stress tolerance pathways. Advanced omics tools can also be employed to profile microbial functions in the soil rhizosphere, shedding light on active metabolic pathways that support plant health. The development of microbial inoculants, particularly those based on PSB and AMF, should also be pursued to improve plant growth and soil fertility under water scarcity. Additionally, analysis via the Kyoto Encyclopedia of Genes and Genomes (KEGG) pathway and enzyme can further be employed to detect and map the functional roles of microbes in the rhizosphere, enhancing our understanding of their contributions to plant health. Finally, integrating microbial-based strategies into land rehabilitation and sustainable agriculture efforts in arid regions could enhance soil health and plant nutrition, ultimately supporting the conservation and sustainable use of *M. crassifolia* in these ecosystems.

## Figures and Tables

**Table 1:** Statistical results of unprocessed DNA sequencing read data obtained from metagenomic samples of bulk soil (S) and rhizosphere soil (R) associated with the plant *M. crassifolia*. The sequencing data were generated from libraries with an average insert size of 350 base pairs, representing the initial metagenomic reads.

Sample ID	Raw Data (Gb) ^1^	Raw Reads (no.)	Low Q (%) ^2^	Ns (%) ^3^	Clean Data (Gb) ^4^	Clean (Q20) (%) ^5^	Clean (Q30) (%) ^6^	Effective (%) ^7^
R1	6183.59	41,223,928	0	0.01	6180.60	97.64	93.97	99.952
R2	6122.59	40,817,252	0	0.01	6119.24	97.46	93.63	99.945
R3	6306.21	42,041,388	0	0	6303.18	97.5	93.61	99.952
R4	6064.85	40,432,340	0	0.01	6062.61	97.73	94.22	99.963
S1	6632.32	44,215,462	0	0.01	6629.34	97.57	93.9	99.955
S2	6590.39	43,935,962	0	0.01	6587.69	97.53	93.69	99.959
S3	6647.59	44,317,254	0	0	6644.91	97.71	94.15	99.96
S4	6763.60	45,090,636	0	0.01	6760.97	97.63	94.01	99.961

^1^ Raw data (Gb): unprocessed sequencing data size in gigabases. ^2^ Low Q (%): percentage of raw reads removed due to quality filtering. ^3^ Ns (%): percentage of bases classified as ’N’ (unknown). ^4^ Clean data (Gb): size of the high-quality sequencing data after filtering, in gigabases. ^5^ Clean Q20 (%): percentage of clean data with a base call accuracy of at least 99%. ^6^ Clean Q30 (%): percentage of clean data with a base call accuracy of at least 99.9%. ^7^ Effective (%): percentage of clean data compared to the raw sequencing data.

## Data Availability

The data presented in this study have been deposited in the National Center for Biotechnology Information (NCBI) repository at https://www.ncbi.nlm.nih.gov/ (accessed on 11 November 2024) under the project accession number PRJNA1181874.

## Supplementary Materials

The following supporting information can be downloaded at: https://www.mdpi.com/article/10.3390/genes16030285/s1. Figure S1. Non-redundant genes count in *M. crassifulia* rhizosphere and bulk soil metagenomes. Figure S2. Rarefaction analysis of the core and pan-genomes revealed divergent trends. Figure S3. Taxonomic classification of metagenomes from bulk (S) and rhizosphere (R) soil samples of the *M. crassifolia* plant, based on non-redundant genes at the domain level. Figure S4. The relative microbial abundance of the top 10 bacterial phyla, based on nRG identified in the metagenomic data of the *M. crassifolia* plant across different soil types (e.g., rhizosphere and bulk). Figure S5. The relative microbial abundance of the top 10 bacterial genera, based on nRG identified in the metagenomic data of the *M. crassifolia* plant across different soil types (e.g., rhizosphere and bulk). Figure S6. The relative microbial abundance of the top 10 bacterial species, based on nRG identified in the metagenomic data of the *M. crassifolia* plant across different soil types (e.g., rhizosphere and bulk). Figure S7. The relative microbial abundance of the top 10 fungal phyla, based on nRG identified in the metagenomic data of the *M. crassifolia* plant across different soil types (e.g., rhizosphere and bulk). Figure S8. The relative microbial abundance of the top 10 fungal genera, based on nRG identified in the metagenomic data of the *M. crassifolia* plant across different soil types (e.g., rhizosphere and bulk). Figure S9. The relative microbial abundance of the top 10 fungal species, based on nRG identified in the metagenomic data of the *M. crassifolia* plant across different soil types (e.g., rhizosphere and bulk). Table S1. Statistical results of assembly for metagenomic sequencing data derived from bulk (S) and rhizosphere (R) soil samples collected near M. crassifolia plants. Table S2. Statistical results of gene prediction for microbiome samples from bulk soil (S) and rhizosphere (R) of the *M. crassifolia* plant. Open reading frames (ORFs) were predicted to identify unique genes.
