# Supplementary material for: Metagenomic Characterization of the Maerua crassifolia Soil Rhizosphere: Uncovering Microbial Networks for Nutrient Acquisition and Plant Resilience in Arid Ecosystems
Source: Genes (Basel). 2025 Feb 26;16(3):285. doi: 10.3390/genes16030285 (PMC11942469; doi:10.3390/genes16030285)
Supplement: Supplementary file 1 [file genes-16-00285-s001.zip › genes-3467045-supplementary.pdf]

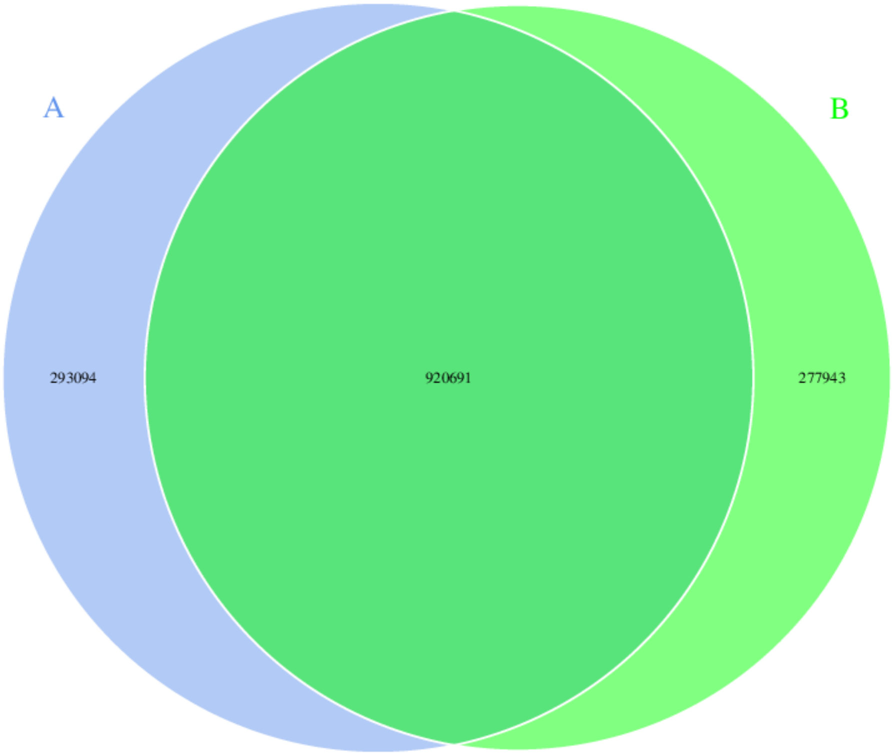

Figure S1. Non-redundant genes count in *M. crassifulia* rhizosphere and bulk soil metagenomes. The Venn diagram depicts the number of non-redundant genes identified in each soil type. Group A (blue circle) represents the rhizosphere soil metagenome, containing 293,094 non-redundant genes. Group B (green circle) represents the bulk soil metagenome, containing 277,943 non-redundant genes. The intersection (dark green circle) represents the 92,069 non-redundant genes shared between both metagenomes.

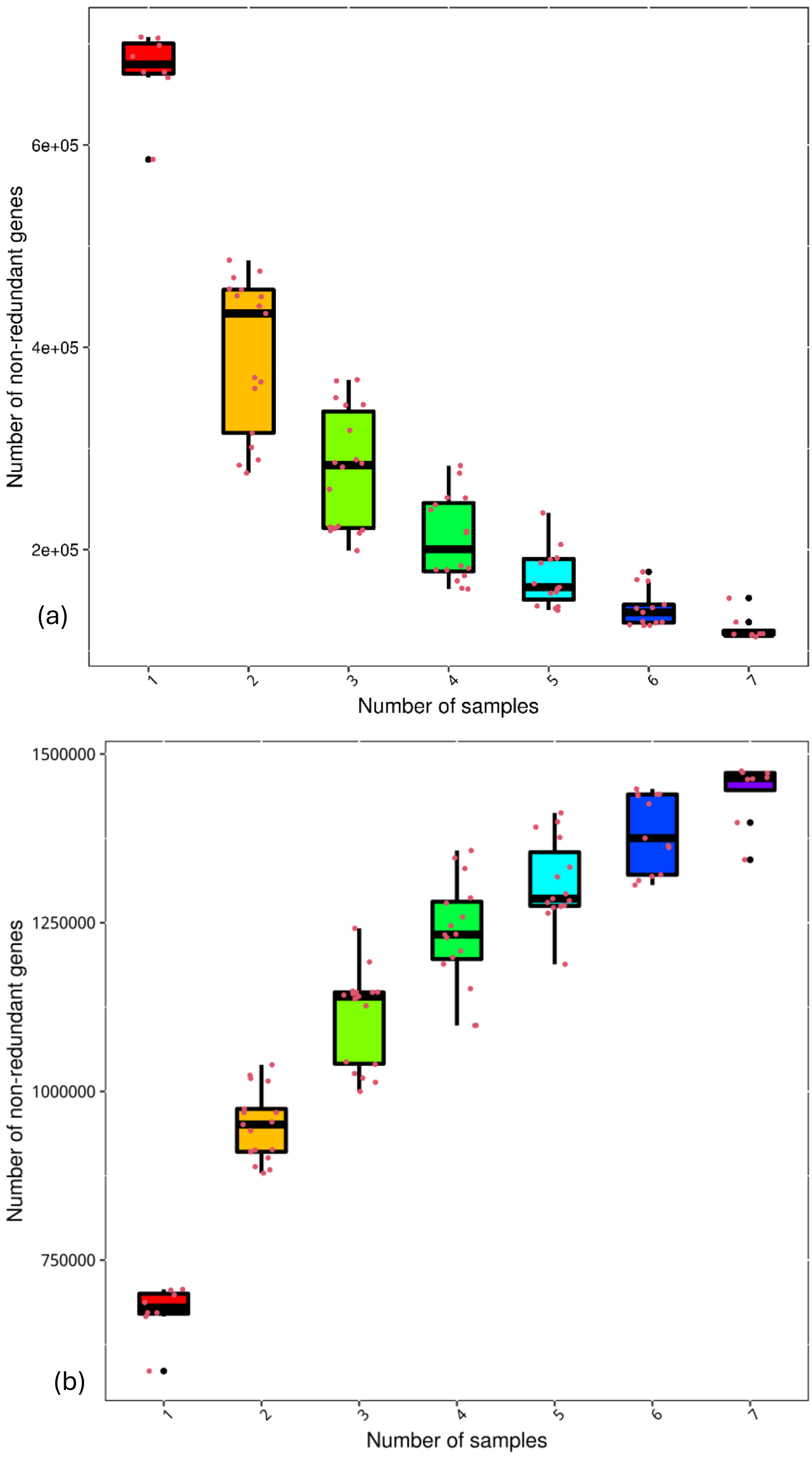

Figure S2. Rarefaction analysis of the core and pan-genomes revealed divergent trends. The core genome, which includes genes found in all samples, showed a diminishing trend with the addition of more samples, indicating a decrease in conserved genes as sample variety rose. In contrast, the pan-genome, which represents the cumulative gene repertoire containing unique genes, showed a rising trend with sample size, showing that the dataset's overall genetic diversity was expanding. The y-axis shows the number of non-redundant genes, while the x-axis represents the number of samples.

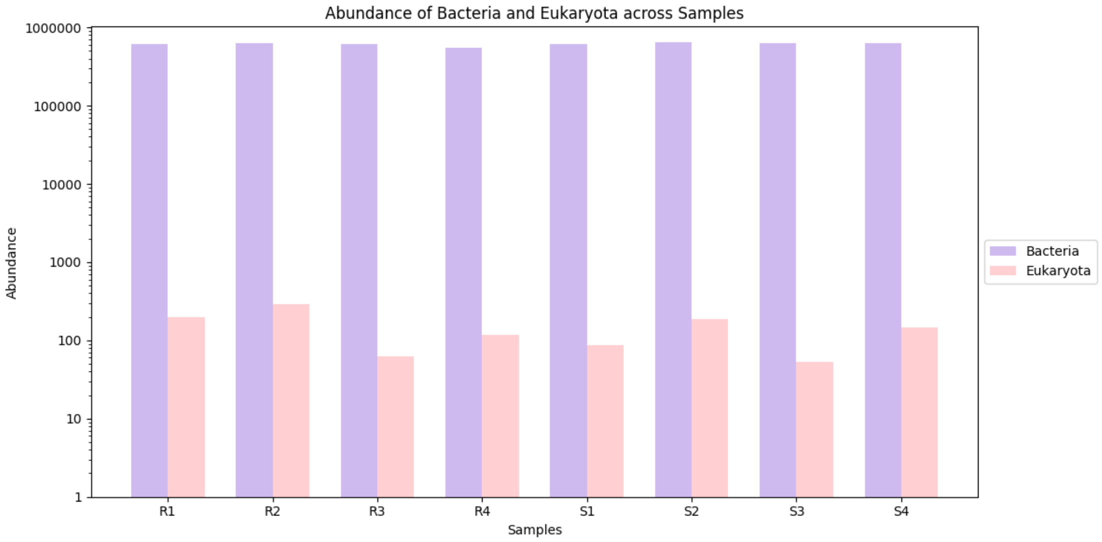

Figure S3. Taxonomic classification of metagenomes from bulk (S) and rhizosphere (R) soil samples of the *M. crassifolia* plant, based on non-redundant genes at the domain level.

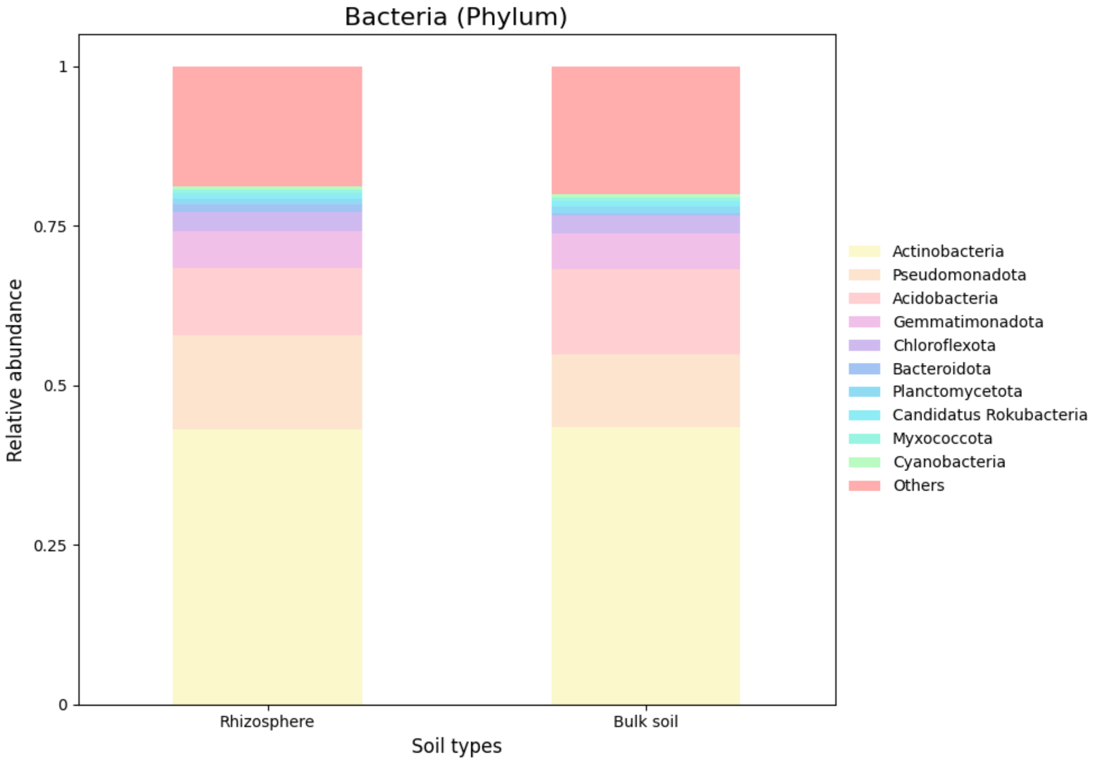

Figure S4. The relative microbial abundance of the top 10 bacterial phyla, based on nRG identified in the metagenomic data of the *M. crassifolia* plant across different soil types (e.g., rhizosphere and bulk).

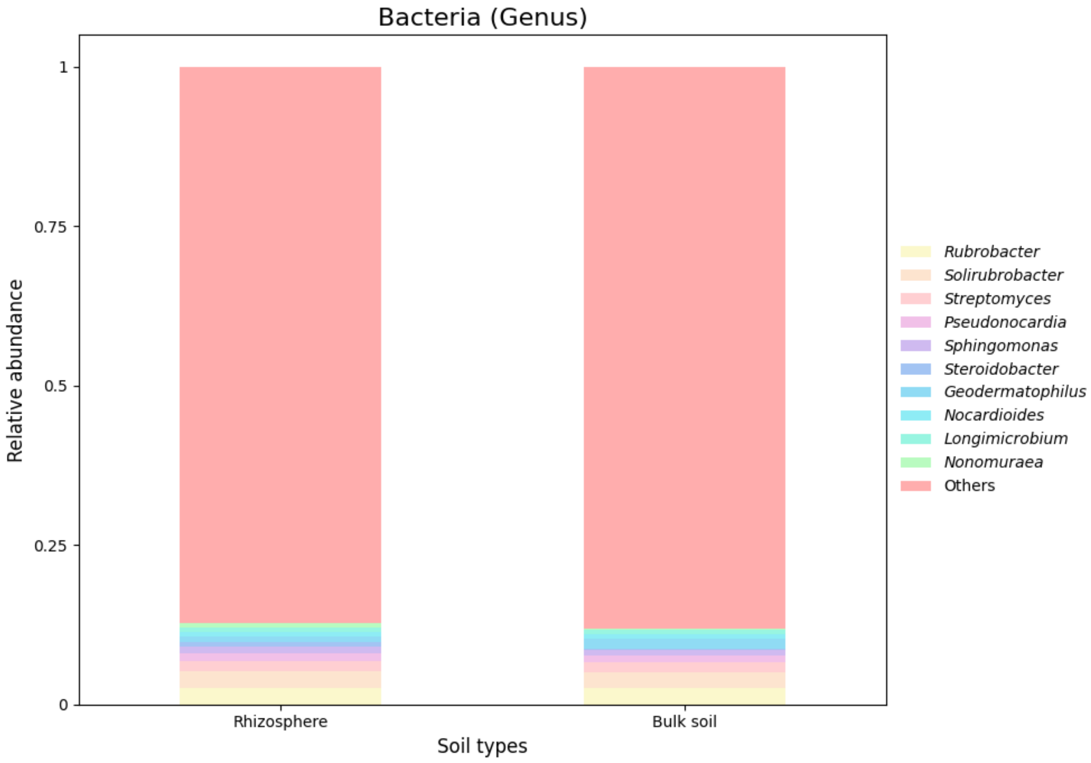

Figure S5. The relative microbial abundance of the top 10 bacterial genera, based on nRG identified in the metagenomic data of the *M. crassifolia* plant across different soil types (e.g., rhizosphere and bulk).

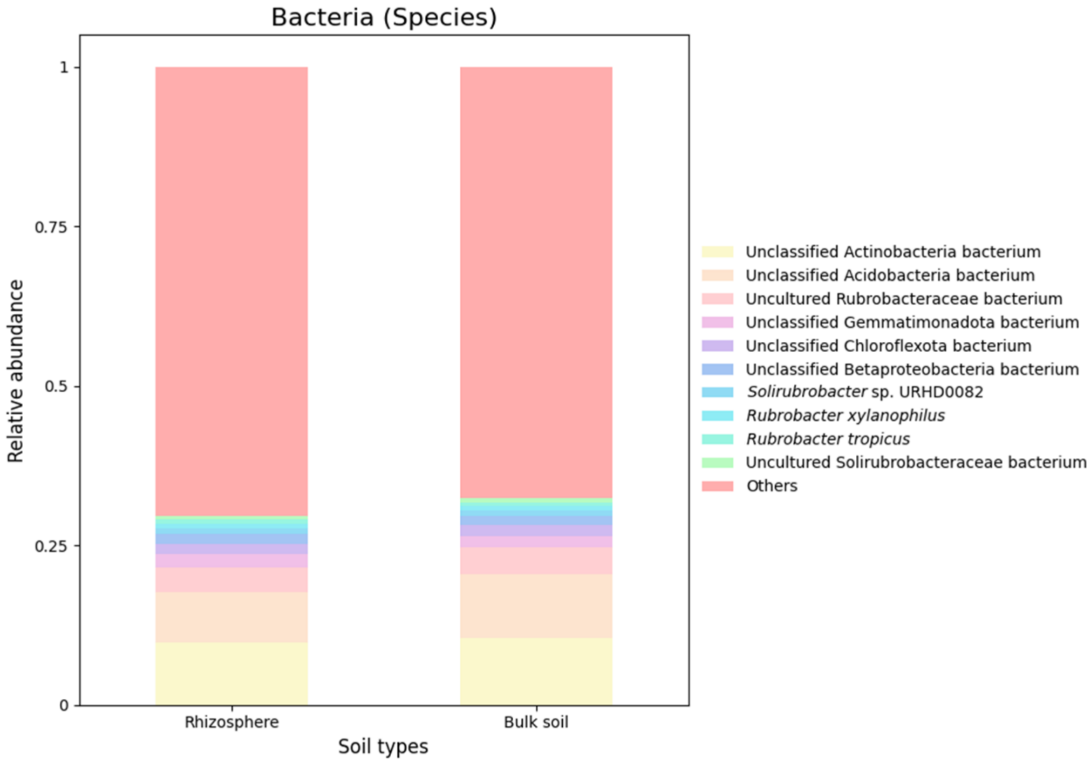

Figure S6. The relative microbial abundance of the top 10 bacterial species, based on nRG identified in the metagenomic data of the *M. crassifolia* plant across different soil types (e.g., rhizosphere and bulk).

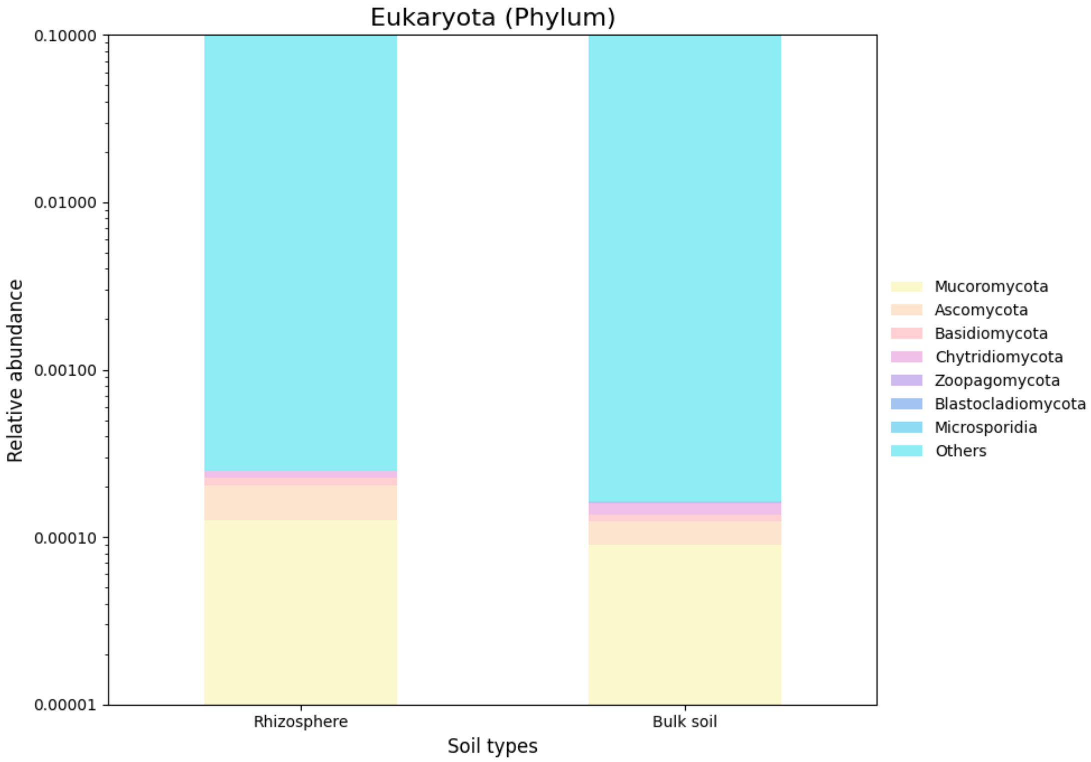

Figure S7. The relative microbial abundance of the top 10 fungal phyla, based on nRG identified in the metagenomic data of the *M. crassifolia* plant across different soil types (e.g., rhizosphere and bulk).

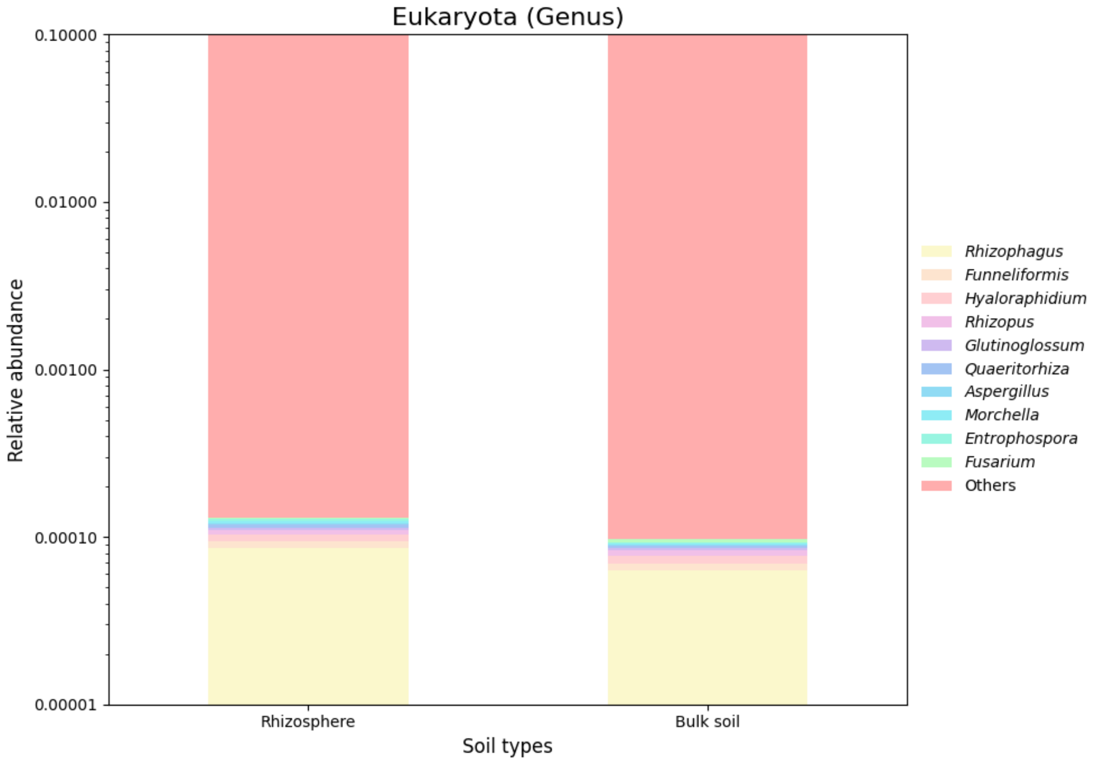

Figure S8. The relative microbial abundance of the top 10 fungal genera, based on nRG identified in the metagenomic data of the *M. crassifolia* plant across different soil types (e.g., rhizosphere and bulk).

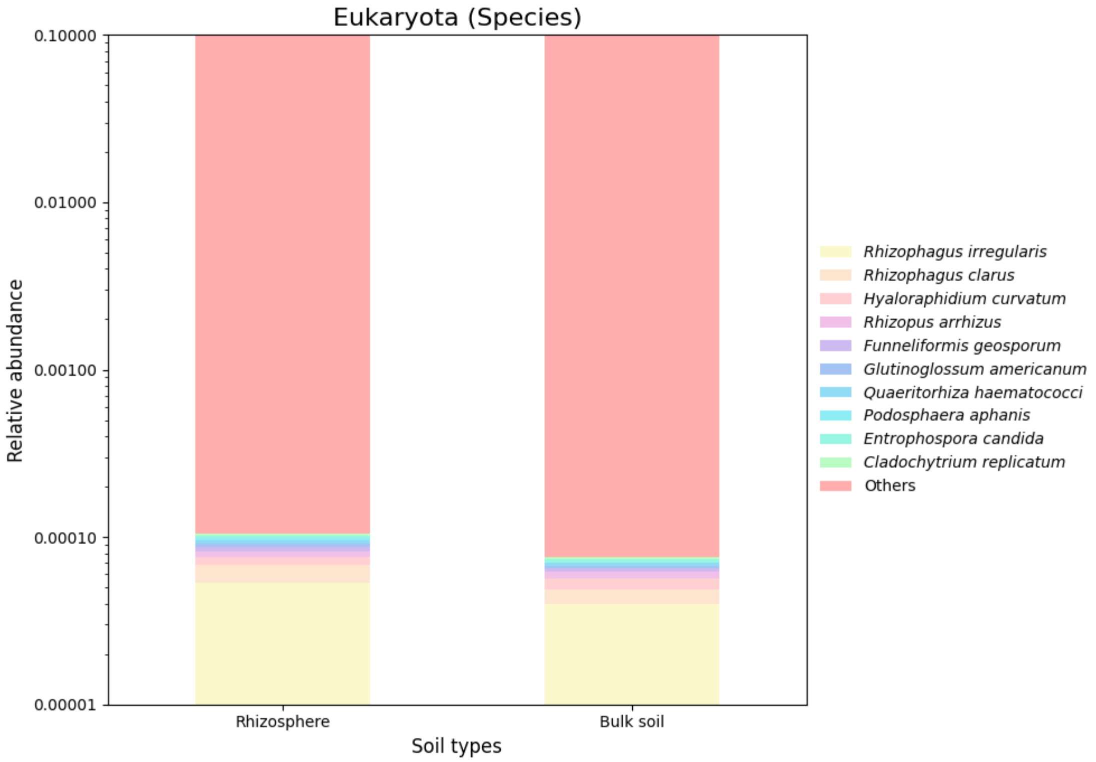

Figure S9. The relative microbial abundance of the top 10 fungal species, based on nRG identified in the metagenomic data of the *M. crassifolia* plant across different soil types (e.g., rhizosphere and bulk).

Table S1. Statistical results of assembly for metagenomic sequencing data derived from bulk (S) and rhizosphere (R) soil samples collected near *M. crassifolia* plants.

Sample ID	Total len. (bp)[1]	No. scaftigs[2]	Average len. (bp)[3]	N50 len. (bp)[4]	N90 len. (bp)[5]	Max len. (bp)[6]
**R1**	112,613,869	132,068	852.7	790	536	217,024
**R2**	103,861,482	138,526	749.76	711	529	47,366
**R3**	110,431,192	141,566	780.07	717	531	163,064
**R4**	164,501,426	177,179	928.45	906	547	59,678
**S1**	85,856,802	122,449	701.16	665	523	9,950
**S2**	101,317,087	134,269	754.58	711	530	21,211
**S3**	100,948,980	133,251	757.59	704	528	44,962
**S4**	170,097,356	187,719	906.13	834	538	208,173
**NOVO_MIX[7]**	130,813,273	206,734	632.76	608	516	3,906

[*1]Total len. (bp): The sum of base pairs across all assembled scaftigs.

[2] No. scaftigs: The count of scaftigs generated during the assembly process.

[3]Average len. (bp): Average length of the scaftigs.

[4] N50 len. (bp): The scaftig length at which 50% of the total assembly length is reached when scaftigs are ordered by size from largest to smallest.

[5] N90 len. (bp): The scaftig length at which 90% of the total assembly length is covered under a similar ranking.

[6]Max len. (bp): The size in base pairs of the single longest assembled scaffold.

[7]NOVO_MIX: Results from a mixed assembly.

Table S2. Statistical results of gene prediction for microbiome samples from bulk soil (S) and rhizosphere (R) of the *M. crassifolia* plant. Open reading frames (ORFs) were predicted to identify unique genes.

Sample ID	ORFs NO.[1]	integrity: none[2]	integrity: start[3]	integrity: end[4]	integrity: all[5]	Total len. (Mbp)[6]	Average len. (bp)[7]
**R1**	190,447	33,566 (17.62%)	65,454 (34.37%)	46,846 (24.6%)	44,581 (23.41%)	96.05	504.33
**R2**	186,006	40,775 (21.92%)	66,998 (36.02%)	46,916 (25.22 %)	31,317 (16.84%)	90.47	486.4
**R3**	200,429	42,136 (21.02%)	71,088 (35.47%)	50,309 (25.1%)	36,896 (18.41%)	98.64	492.13
**R4**	274,068	46,122 (16.83%)	96,572 (35.24%)	68,698 (25.07 %)	62,676 (22.87%)	147.16	536.96
**S1**	162,250	37,063 (22.84%)	59,478 (36.66%)	41,366 (25.5%)	24,343 (15%)	75.6	465.98
**S2**	178,786	41,135 (23.01%)	63,346 (35.43%)	44,955 (25.14 %)	29,350 (16.42%)	88.59	495.53
**S3**	184,316	39,095 (21.21%)	65,168 (35.36%)	46,779 (25.38 %)	33,274 (18.05%)	88.4	479.63
**S4**	287,251	54,828 (19.09%)	96,090 (33.45%)	70,341 (24.49 %)	65,992 (22.97%)	153.03	532.73
**NOVO_MIX**	259,169	66,980 (25.84%)	95,638 (36.9%)	64,677 (24.96 %)	31,874 (12.3%)	115.18	444.44

[*1]ORFs No.: Number of unique genes identified.

[2]Integrity: none: Number and percentage of genes missing both start and stop codons.

[3]Integrity: start: Number and percentage of genes with a start codon but missing a stop codon.

[4]Integrity: end: Number and percentage of genes with a stop codon but missing a start codon.

[5]Integrity: all: Number and percentage of genes containing both start and stop codons.

[6]Total len. (Mbp): Combined length of all identified genes.

[7]Average len.: Average length of individual genes.
